# Supplementary material for: Genetic association and transferability for urinary albumin-creatinine ratio as a marker of kidney disease in four Sub-Saharan African populations and non-continental individuals of African ancestry
Source: Front Genet. 2024 May 15;15:1372042. doi: 10.3389/fgene.2024.1372042 (PMC11134365; doi:10.3389/fgene.2024.1372042)
Supplement: Supplementary file 2 [file Image1.PDF]

Genetic Association and Transferability for Urinary Albumin-Creatinine Ratio as a Marker of Kidney Disease in four Sub-Saharan African Populations and non-continental Individuals of African Ancestry

Jean-Tristan Brandenburg

April 4, 2024

List of Figures

1 Genetic Diversity of pooled samples using PC1 and 2 3
2a QQ plot and lambda value for UKB-African, UKB-Caribbean, AWI-Agincourt, ARK-Agincourt, AWI-Dikgale, AWI-Navrongo, AWI-Nanoro, AWI-Soweto and AWI-Nairobi 4
2b QQ plot and lambda value for Meta_{SSA}, Meta_{ALL} and $\text{Meta}_{\text{NONRES}}$ 4
3a Manhattan plot for UKB-African 5
3b Manhattan plot for UKB-Caribbean 5
3c Manhattan plot for ARK-Agincourt 6
3d Manhattan plot for AWI-Agincourt 6
3e Manhattan plot for AWI-Dikgale 7
3f Manhattan plot for AWI-Nanoro 7
3g Manhattan plot for AWI-Navrongo 8
3h Manhattan plot for AWI-Soweto 8
3i Manhattan plot for AWI-Nairobi 9
4a Regional plot around rs2052976 (20-13014686) using AWI-Dikgale results 10
4b Regional plot around rs147938214 (2-147380678) using AWI-Soweto results 11
4c Regional plot around rs12067862 (1-232,389,864) using UKB-Caribbean results 12
5 Forest plot (beta, se) and af, p-value and N for each dataset and meta analysis result of rs9505286 (chr6,7820353) found significant in Meta_{SSA} 13

6 Forest plot (beta, se) and af, p-value and N for each dataset and meta analysis of rs73404549 (chr11,5320654) found significant in $\text{Meta}_{\text{NONRES}}$, Meta_{ALL} 14
7 Replication of *THBS3* regions identified in CKDGEN-EA : forest plot of lead SNPs identified in Meta_{SSA} rs370545 (a) and regional plot around (b) 15
8 Replication of *ARL15* regions identified in CKDGEN-EA and CKDGEN-MA : forest plot of lead SNPs identified in Meta_{SSA} rs1694067 (a) and regional plot around (b) 16
9 replication of *GATM/SPATA5L1* regions identified in CKDGEN-EA : forest plot of lead SNPs identified in Meta_{ALL} rs1153847 (15-45696890) (a) and regional plot around (b) 17
10a Manhattan plot of Meta Analysis using South resident using Fixed effect method 18
10b Manhattan plot of Meta Analysis using West resident using Fixed effect method 19
11 Manhattan plot of Meta Analysis using all dataset using Random effect 20

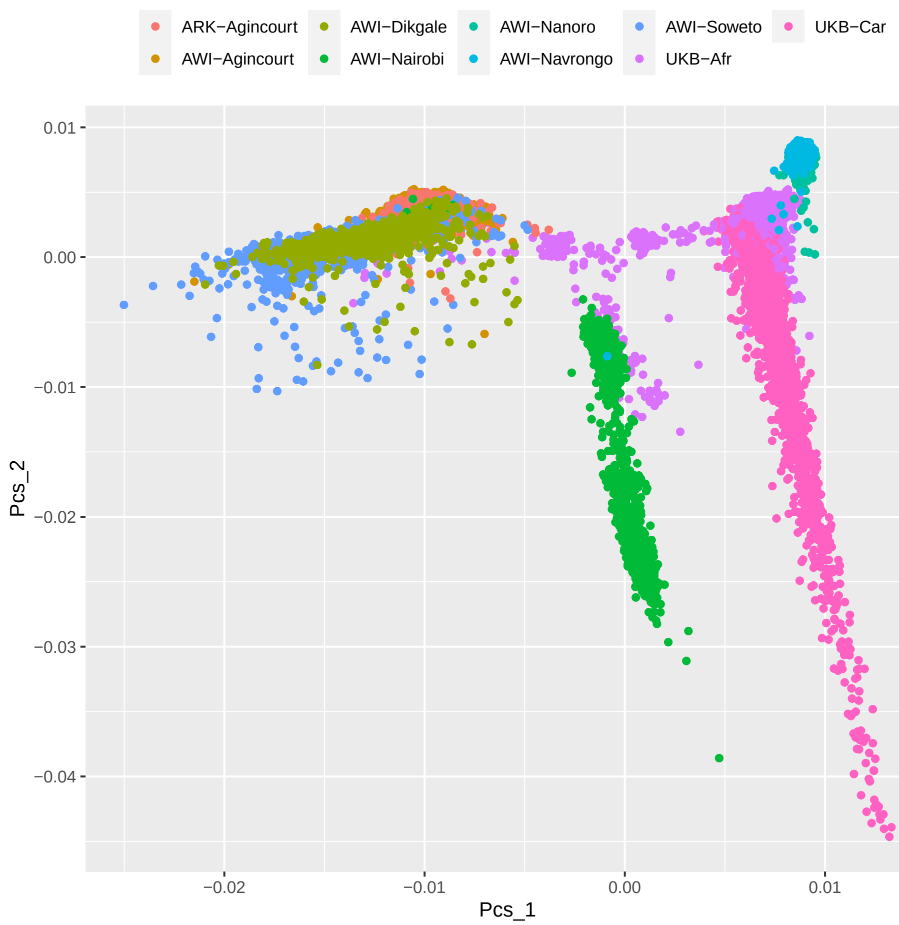

Figure S1: Genetic Diversity of pooled samples using PC1 and 2

AWIGEN: Africa Wits-INDEPTH partnership for Genomic Studies ; ARK: African Research on Kidney Disease Study;UKB: UK Biobank

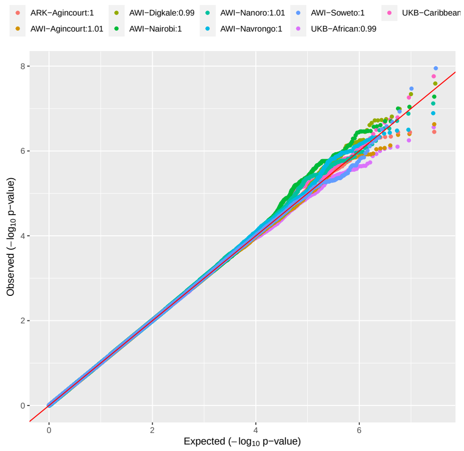

Figure S2a: QQ plot and lambda value for UKB-African, UKB-Caribbean, AWI-Agincourt, ARK-Agincourt, AWI-Dikgale, AWI-Navrongo, AWI-Nanoro, AWI-Soweto and AWI-Nairobi

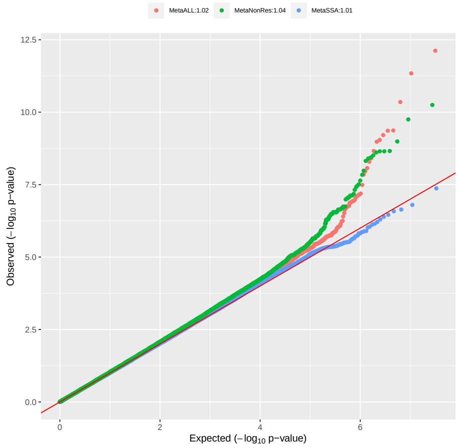

Figure S2b: QQ plot and lambda value for Meta_{SSA}, Meta_{ALL} and $\text{Meta}_{\text{NONRES}}$

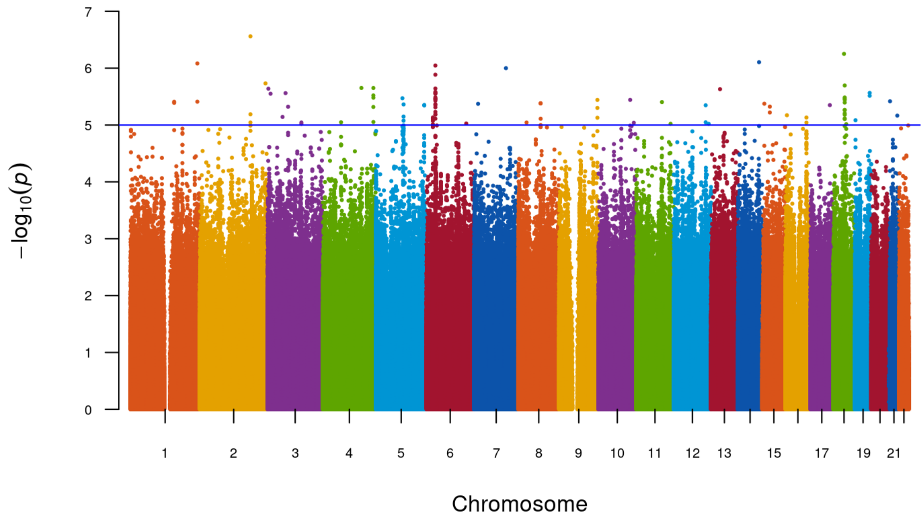

Figure S3a: Manhattan plot for UKB-African

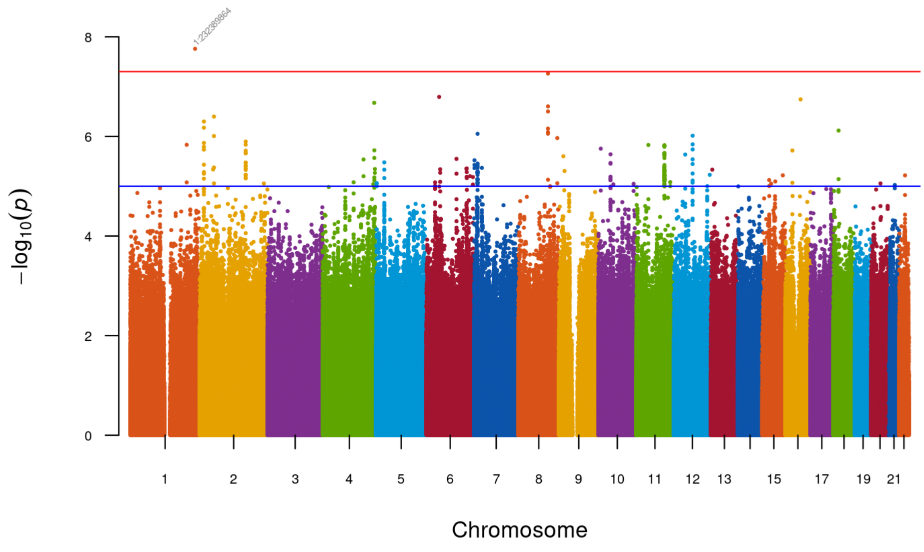

Figure S3b: Manhattan plot for UKB-Caribbean

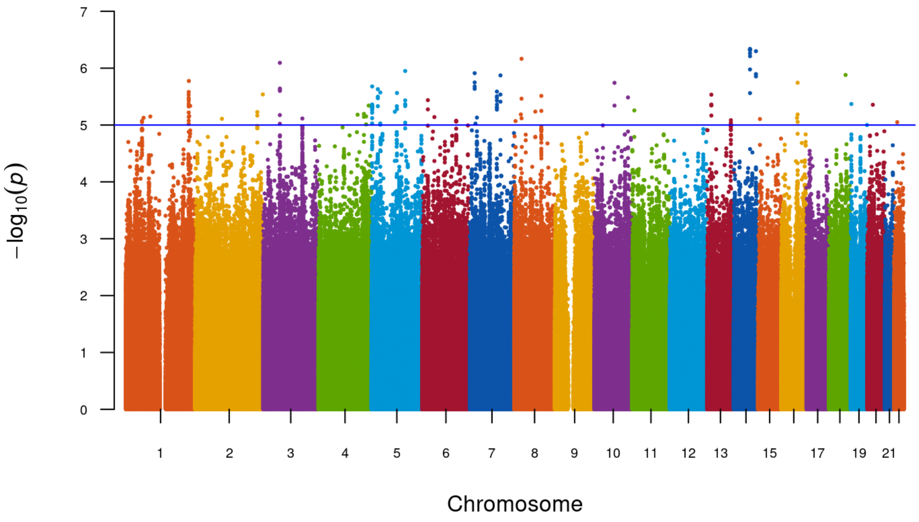

Figure S3c: Manhattan plot for ARK-Agincourt

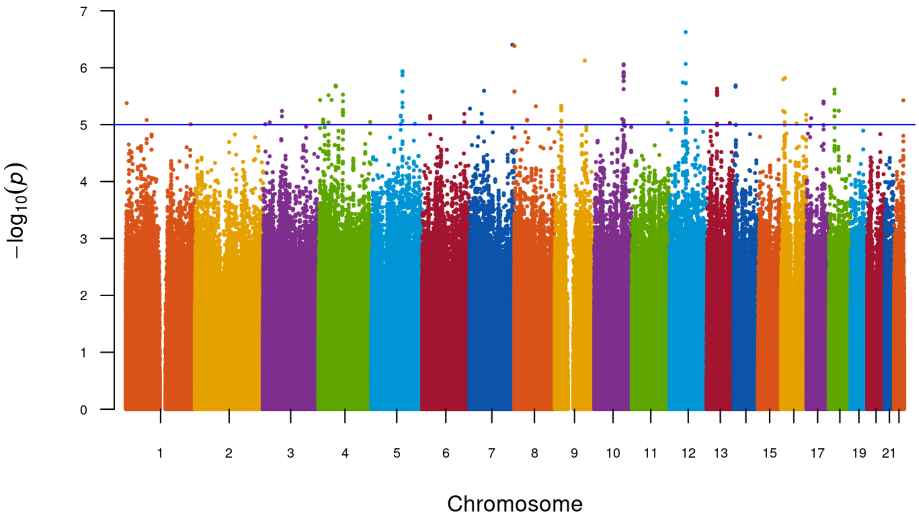

Figure S3d: Manhattan plot for AWI-Agincourt

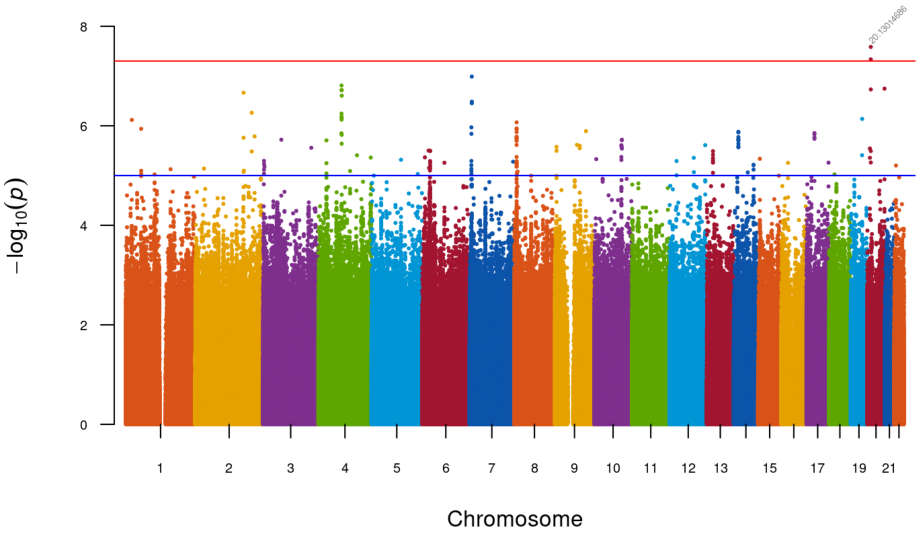

Figure S3e: Manhattan plot for AWI-Dikgale

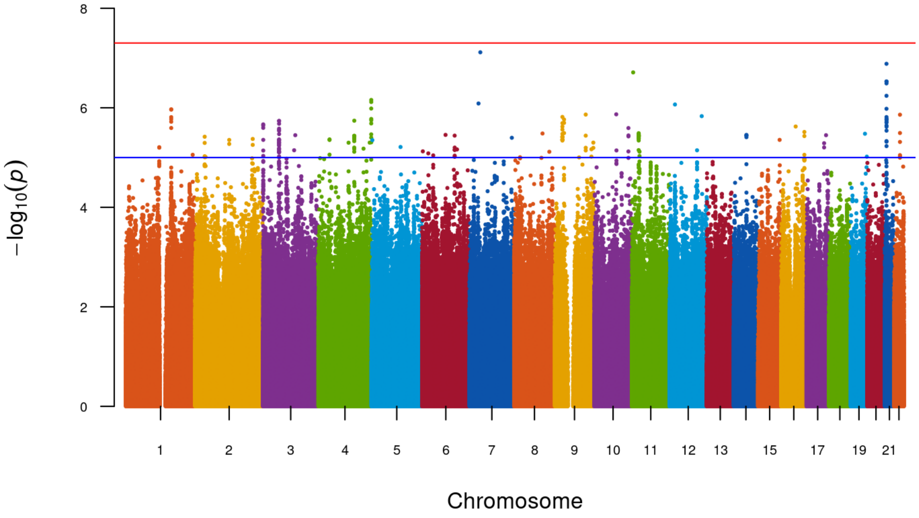

Figure S3f: Manhattan plot for AWI-Nanoro

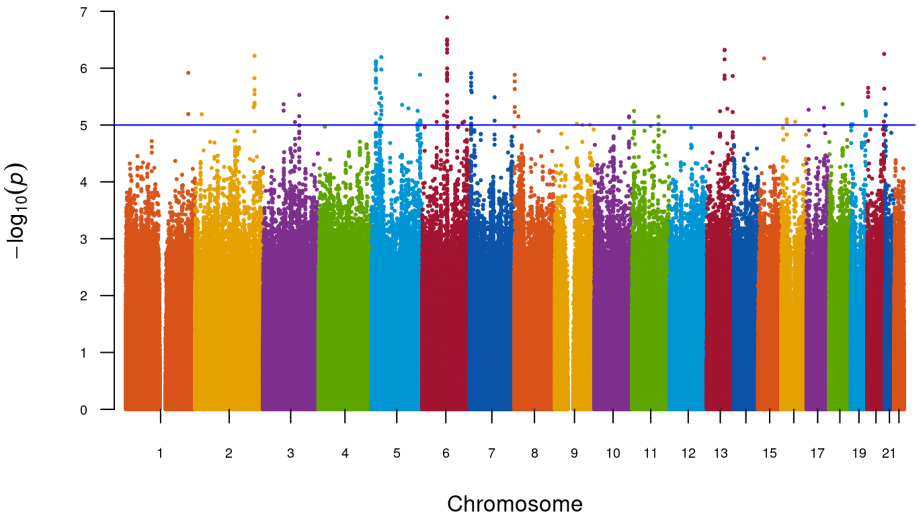

Figure S3g: Manhattan plot for AWI-Navrongo

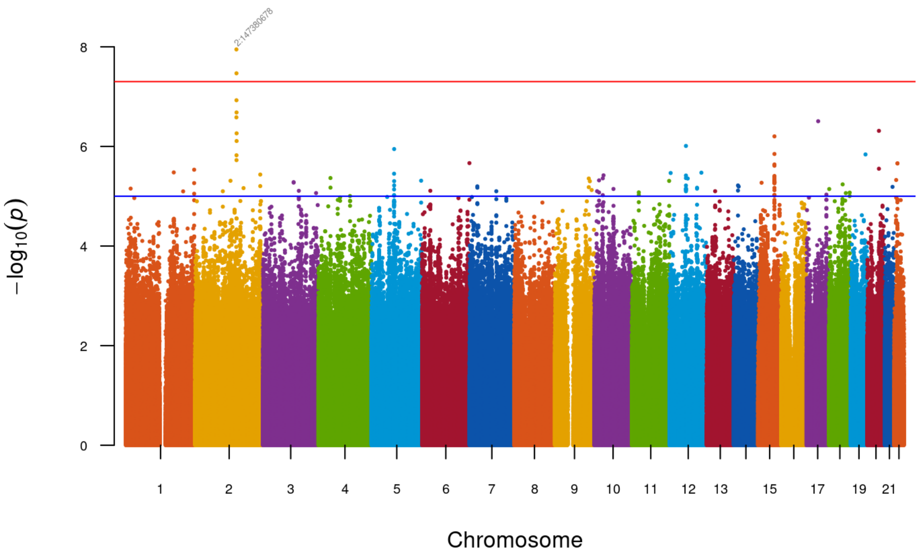

Figure S3h: Manhattan plot for AWI-Soweto

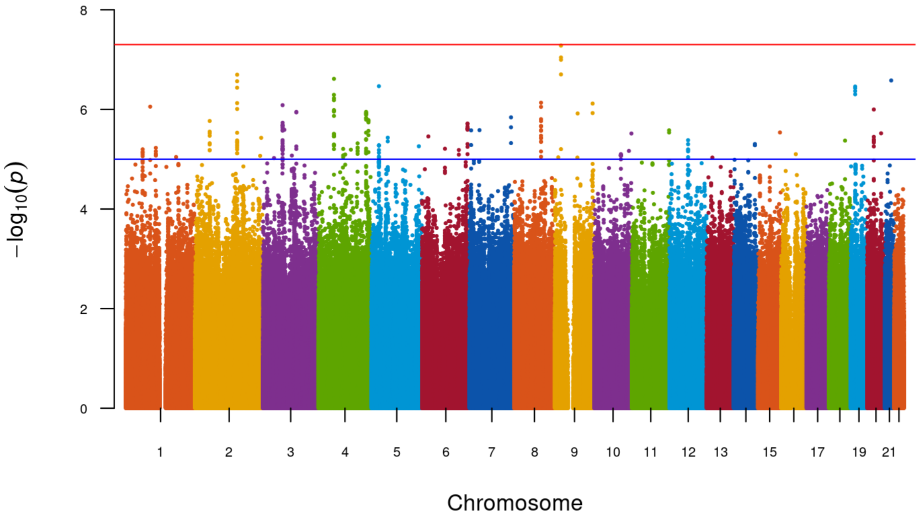

Figure S3i: Manhattan plot for AWI-Nairobi

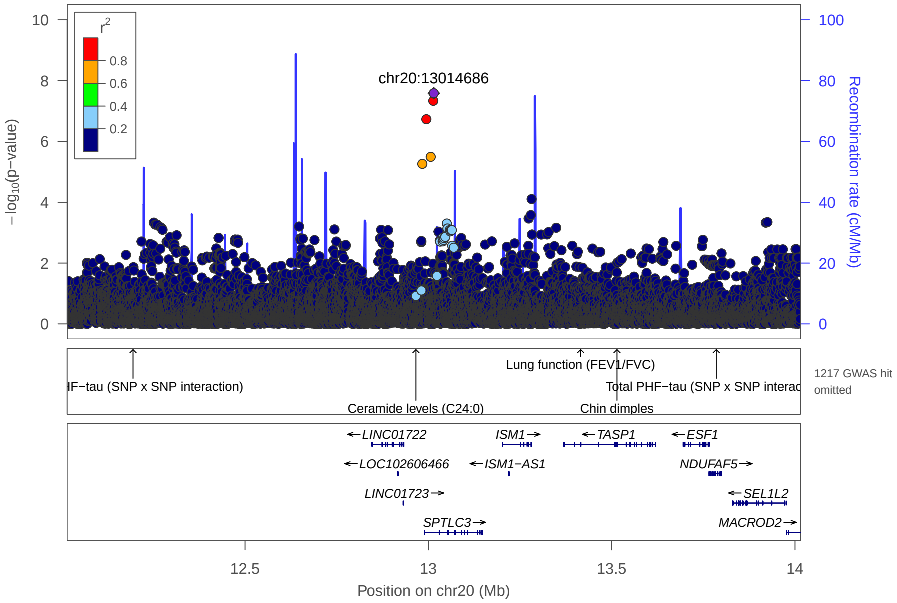

Figure S4a: Regional plot around rs2052976 (20-13014686) using AWI-Dikgale results

rs2052976 found significant in AWI-Dikgale, regional plot had been done using locuszoom with a windows of 1MB around and LD had been estimated using sample from AWI-Dikgale

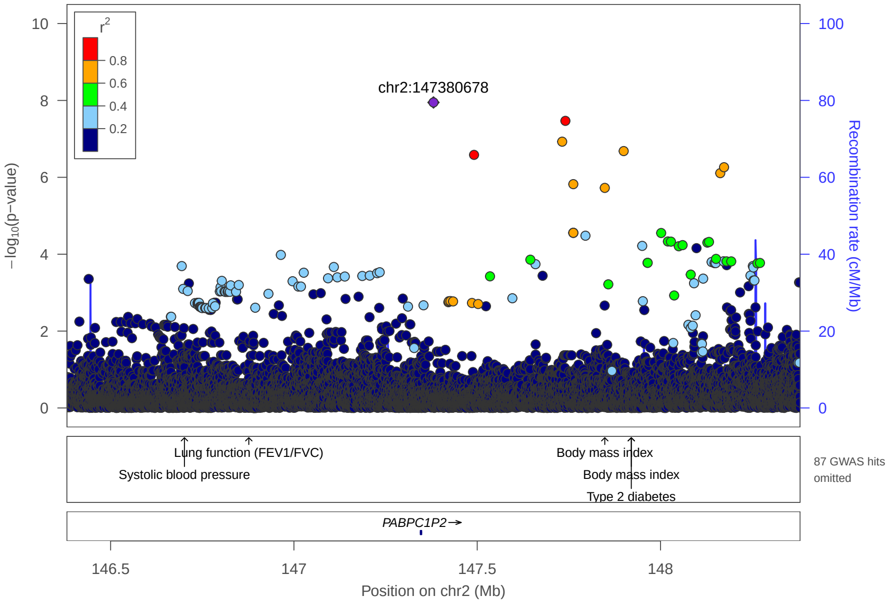

Figure S4b: Regional plot around rs147938214 (2-147380678) using AWI-Soweto results

rs147938214 found significant in AWI-Soweto, regional plot had been done using locuszoom with a windows of 1MB around and LD had been estimated using sample from AWI-Soweto

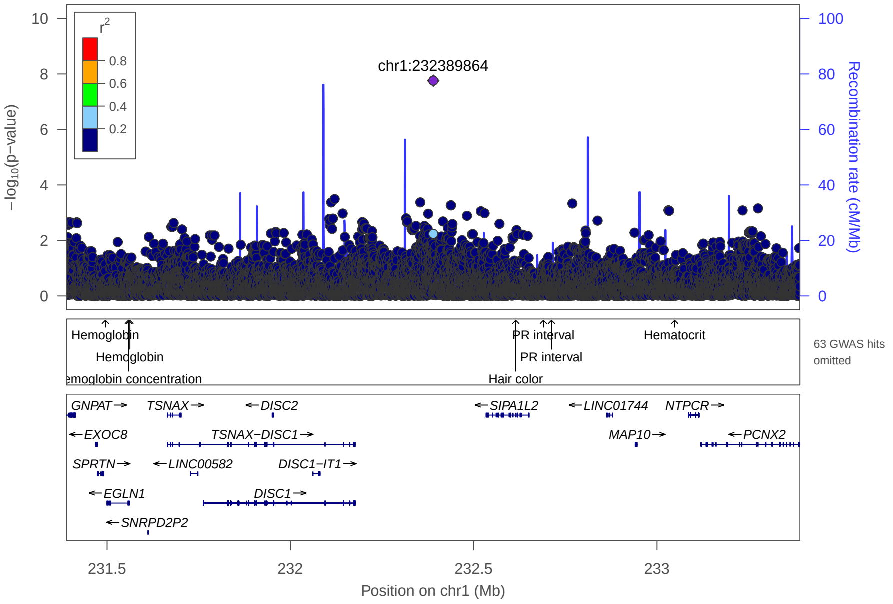

Figure S4c: Regional plot around rs12067862 (1-232,389,864) using UKB-Caribbean results

rs12067862 found significant in UKB-Caribbean, regional plot had been done using locuszoom with a windows of 1MB around and LD had been estimated using sample from UKB-Caribbean

Study	af	P	N
Ark−Agincourt	0.945	0.13	928
AWI−Agincourt	0.951	3.4e−03	1545
AWI−Soweto	0.965	0.17	766
AWI−Dikgale	0.967	0.25	917
AWI−Nanoro	0.943	0.01	1702
AWI−Navrongo	0.961	7.0e−03	1548
AWI−Nairobi	0.937	0.06	1481
**MetaSSA**	**0.951**	**4.3e−08**	**8887**
UKB−African	0.949	0.1	1204
UKB−Caribbean	0.956	0.09	1710
CKDGEN−AA	0.966	0.11	6795
**MetaNONRES**	**0.962**	**0.17**	**9709**
**MetaIALL**	**0.957**	**7.2e−07**	**18596**

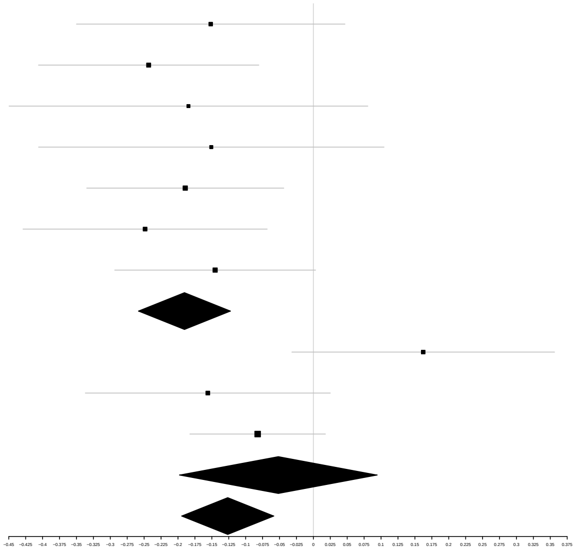

Figure S5: Forest plot (beta, se) and af, p-value and N for each dataset and meta analysis result of rs9505286 (chr6,7820353) found significant in Meta_{SSA}

Study	af	P	N
Ark−Agincourt			
AWI−Agincourt			
AWI−Soweto			
AWI−Dikgale			
AWI−Nanoro	0.04	0.13	1702
AWI−Navrongo	0.053	8.8e−03	1548
AWI−Nairobi			
**MetaSSA**	**0.046**	**3.2e−03**	**3250**
UKB−African	0.089	6.1e−05	1204
UKB−Caribbean	0.053	3.1e−05	1710
CKDGEN−AA	0.046	1.5e−04	6795
**MetaNONRES**	**0.053**	**5.6e−11**	**9709**
**MetaALL**	**0.051**	**7.7e−13**	**12959**

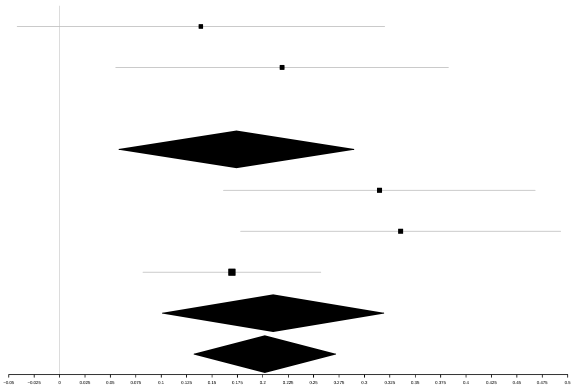

Figure S6: Forest plot (beta, se) and af, p-value and N for each dataset and meta analysis of rs73404549 (chr11,5320654) found significant in $\mathrm{Meta}_{\mathrm{NONRES}}$, $\mathrm{Meta}_{\mathrm{ALL}}$

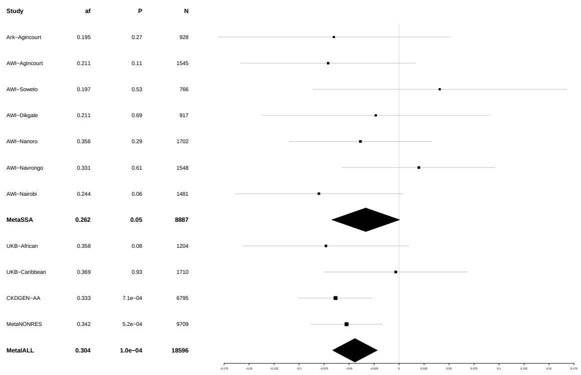

(a) Forest plot of rs370545

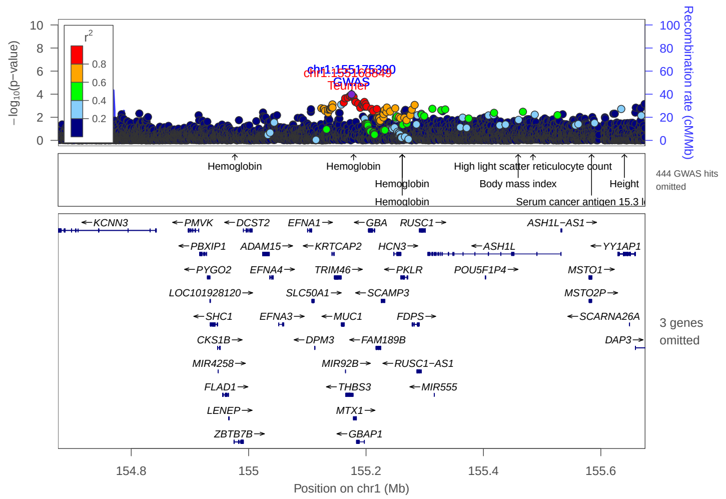

(b) Regional plot around rs370545 in Meta_{ALL}

Figure S7: Replication of *THBS3* regions identified in CKDGEN-EA : forest plot of lead SNPs identified in Meta_{SSA} rs370545 (a) and regional plot around (b)
regional plot had been perfomed using locus zoom, using LD_{ALL}, lead SNPs identified in Meta_{ALL} are annotated in blue and Significant SNPs identified in CKDGEN-EA are highligth in red (b)

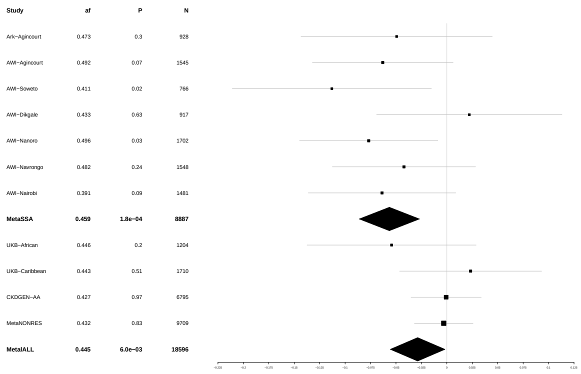

(a) Forest plot of rs1694067

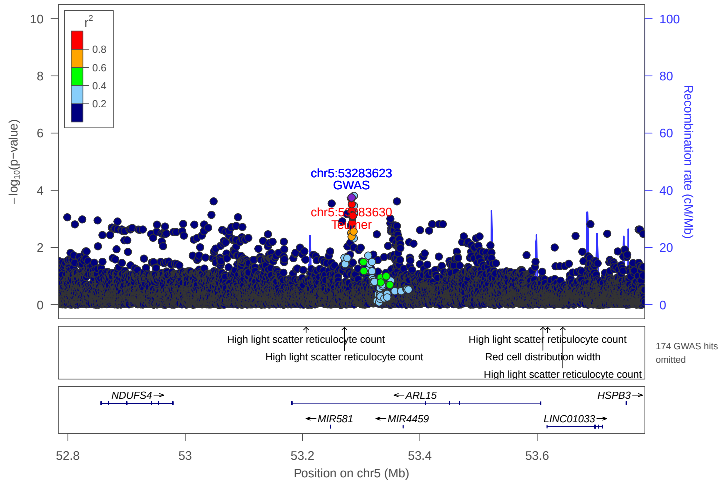

(b) Regional plot using Meta_{SSA} and CKDGEN-MA

Figure S8: Replication of *ARL15* regions identified in CKDGEN-EA and CKDGEN-MA : forest plot of lead SNPs identified in Meta_{SSA} rs1694067 (a) and regional plot around (b)
regional plot had been perforned using locus zoom, using LD_{SSA}, lead SNPs identified in Meta_{SSA} are annotated in blue and Significant SNPs identified in CKDGEN-MA are highligth in red (b)

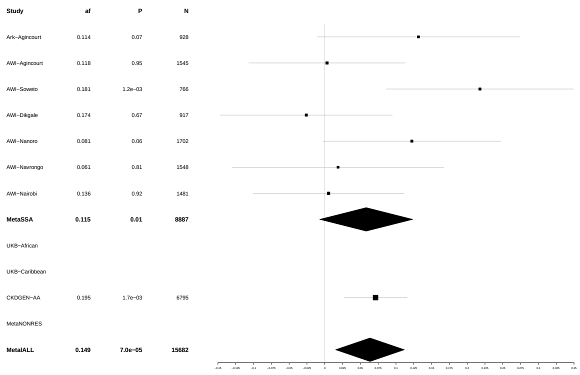

(a) Forest plot of rs1153847

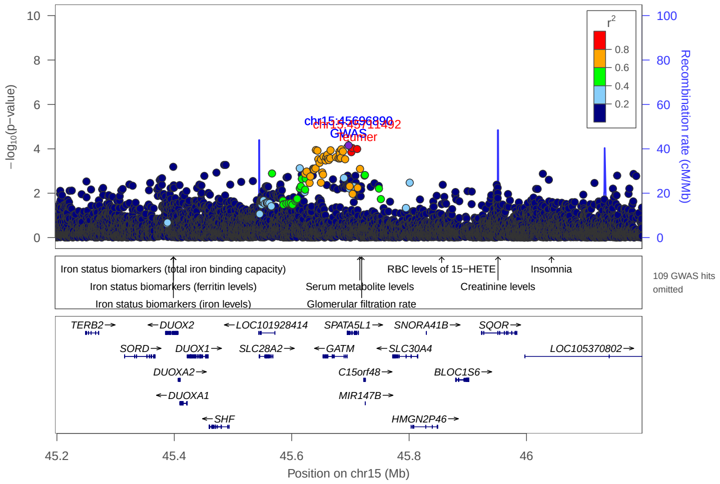

(b) Regional plot around rs1153847 using Meta$_{\mathrm{ALL}}$ and CKD-GEN-EA

Figure S9: replication of *GATM/SPATA5L1* regions identified in CKDGEN-EA : forest plot of lead SNPs identified in Meta$_{\mathrm{ALL}}$ rs1153847 (15-45696890) (a) and regional plot around (b)
regional plot had been perforned using locus zoom, using LD$_{\mathrm{ALL}}$, lead SNPs identified in Meta$_{\mathrm{ALL}}$ are annotated in blue and Significant SNPs identified in CKDGEN-EA are highligth in red (b)

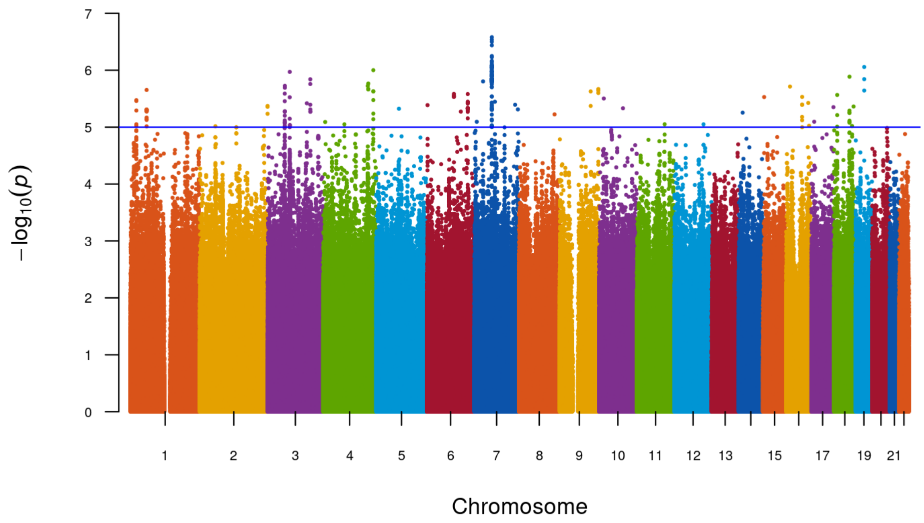

Figure S10a: Manhattan plot of Meta Analysis using South resident using Fixed effect method

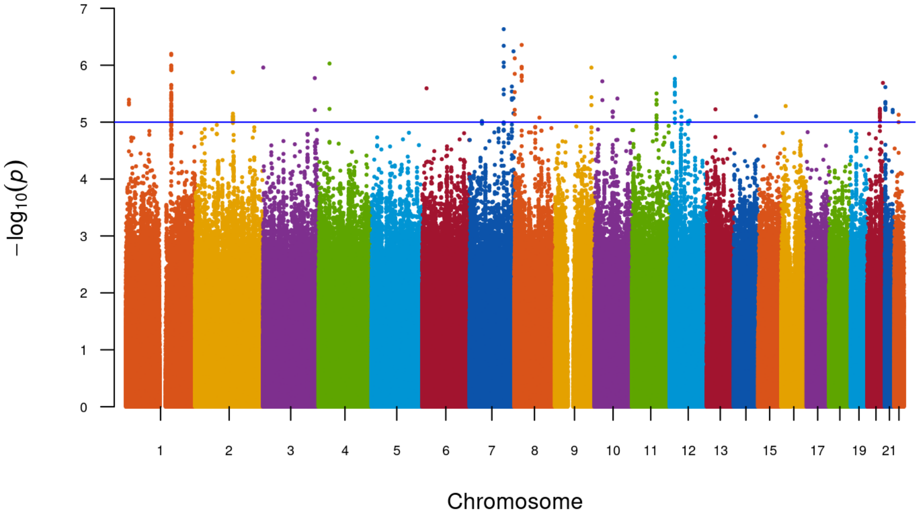

Figure S10b: Manhattan plot of Meta Analysis using West resident using Fixed effect method

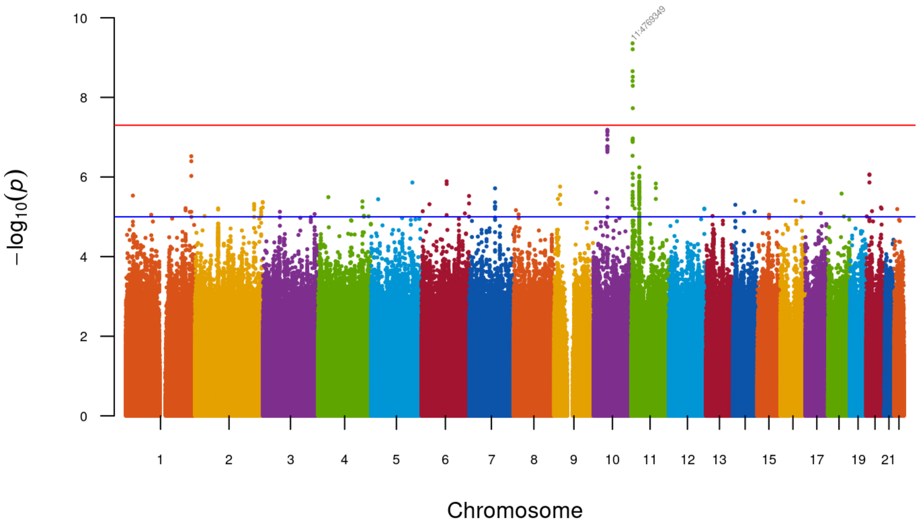

Figure S11: Manhattan plot of Meta Analysis using all dataset using Random effect
